# Pulmonary effects of exposure to pig barn air

**DOI:** 10.1186/1745-6673-1-10

**Published:** 2006-06-06

**Authors:** Chandrashekhar Charavaryamath, Baljit Singh

**Affiliations:** 1 Department of Veterinary Biomedical Sciences and Immunology Research Group, University of Saskatchewan, Saskatoon, SK S7N 5B4, Canada

## Abstract

Swine production has undergone rapid transformation from family owned operation to a large scale industrial enterprise. Since increasing number of pigs are reared on a large scale in confined buildings, some of the swine barn workers may be employed to work eight hours per day. Swine barn workers suffer from higher incidences of impaired air flow and lung inflammation, which is attributed to high intensity and interrupted exposures to pig barn air. The air in these barns contains gases, dust, microbes and endotoxin with endotoxin being the major suspect as the cause of lung dysfunction. This review attempts to describe the current state of knowledge of incidences and mechanisms of pulmonary dysfunction following exposure to the barn air.

## Changing face of pig industry in Canada

Canada is among the top five pork exporters of the world with a total pork export of 970,000 tons in the year 2004, which translates into cash receipts of $4.2 billion in the year 2004 representing a 25% increase over the year 2003. In 2005, Canada had 14.9 million hogs which is an increase of 1.7% over the previous year and the pork export is expected to grow by 2% [1,2]. Currently, pork sector accounts for 30% of total livestock shipments and for 10% of all farm cash receipts in Canadian farm economy. Further, swine farming has provided employment to 10,790 people in Canada [3]. Therefore, swine production is a major component of Canada's agricultural economy. Although the number of pigs has increased but the number of farms has shown a decline to indicate fewer people are working longer shifts on the farms. Now a days, small family operated pig farms are making way for large scale facilities where thousands of pigs are raised in a single facility [4]. Large pig production operations require many full time workers who work 8 hour/day and 5 days/week and thus experience high intensity interrupted exposures to the barn air [5,6]. However, still many workers may work only a few hours every day inside a pig barn. The barn air is very complex and contains organic dust, plant materials (pollen grains, feed grains, hay and silage), animal origin materials (swine dander, hair, urine and pig proteins), microbial components (mite or their parts, bacteria, endotoxin, (1–3) β-D-glucan and fungal spores) and a number of gases such as ammonia, carbon dioxide, hydrogen sulphide and methane [7-9]. Therefore, although modern barns appear cleaner, the air inside these barns still carries toxic molecules which are harmful to the workers [10].

## Clinical symptoms

Exposure to the toxic molecules in the barn air is a risk factor for the development of chronic respiratory symptoms and lung dysfunction [11]. Respiratory diseases in agricultural farmers are one of the earliest recognized occupational hazards [12]. Among the agriculture workers, swine farmers report higher prevalence of occupational respiratory symptoms than in other farmers [13]. Exposed workers report significantly higher frequencies of respiratory symptoms, chest illness, cold and pneumonia [7,14]. The severity of respiratory symptoms in the workers increases during the winter due to the reduced ventilation and is also related to the number of working hours [15].

Previous studies have recorded reductions in expired flow rates in barn workers [11][16-19]. Further, barn workers also exhibit increased airway responsiveness and airway inflammation [20,21]. The longitudinal decline in lung function in swine barn workers has been linked to air contaminants [22] and a dose-response relationship exists between decline in lung function and endotoxin and ammonia levels in the barn air [17]. Exposure to the barn organic dust causes airway inflammation and increased airway resistance both in humans and animal models apart from contributing to the exacerbation of asthma [23-27]. These observations show that the barn air contains toxic molecules which induce lung dysfunction in pig barn workers.

## Effects of acute single exposure to the swine barn air: human studies

To better understand the negative effects of exposure to swine barn air, many researchers have exposed naïve, healthy volunteers to the swine barn air for a short period of time (2–5 hours, once). This study model mimics the lung response of new workers following first exposure to the swine barn air. Single two-five hours of exposure of naïve, healthy volunteers to swine barn air is shown to induce bronchial responsiveness [28], fever, malaise and drowsiness [21]. Across the shift change in lung function during exposure is an important predictor of longitudinal changes in lung function in swine confinement workers [29]. Further, a 75-fold increase in neutrophils, a two-three fold increase in mononuclear cells and a significant increase in eosinophils, fibronectin and albumin levels in bronchoalveolar lavage fluid (BALF) [21] and levels of IL-1β, IL-1 receptor antagonist, IL-6 and TNF-α increased in the serum of the exposed naïve volunteers. The changes in IL-1 receptor antagonist levels correlated with changes in FEV1, bronchial responsiveness, oral temperature and white blood cell count while IL-1β levels correlated with oral temperature [30]. Further, it is shown that, a single exposure to barn air also caused thickening of nasal mucosa, increased numbers of neutrophils in nasal lavage and BALF, increased numbers of macrophages, lymphocytes, eosinophils and the levels of IL-8 in the BALF [31,10]. Interestingly, levels of IL-8, a potent chemoattractant for neutrophils, correlated with increase in neutrophils in the nasal lavage fluid [31]. It is obvious that single exposure to the barn air can activate an inflammatory response in human lungs.

## Animal exposure studies

Although data from studies involving human volunteers has shown induction of lung inflammation following exposure to the barn air, animal studies are critically needed to better understand cell and molecular changes. So far, there have been very few animal model studies to map the mechanisms of barn air-induced lung dysfunction. For example, rabbits and guinea pigs maintained for 12 months in a confined nursery-grower unit showed diffuse interstitial histiocytic pneumonia, epithelial hyperplasia and metaplasia of tracheal and nasal turbinates, with sub mucosal infiltration of plasma cells and heterophils [32]. Interestingly, blood from these test animals contained serum precipitins to dust extract from the swine confinement building [32].

To mimic the work schedule at modern pig barns, we exposed Sprague-Dawley rats to pig barn air for a period of 8 hours/day for one day, 5 days or 20 days. The exposures were interrupted for 2 days after each 5 days of 8 hour/day exposure and rats were kept in clean air during this interruption. Rats exposed to the swine barn air for one or five times showed increase in airway hyperresponsiveness compared to those exposed 20 times or controls. Lungs from all the three exposed groups (one, five and 20 day) were inflamed with recruitment of neutrophils and eosinophils in five and 20 day exposed groups. [33]. These responses are similar to those observed in human volunteers exposed to the barn air for 3–5 hours [28,34,35]. Interestingly, airway reactivity in rats following 20 exposures was not different compared to the controls to suggest physiological adaptation to the pig barn air. Similarly, following single exposure to the barn air, swine farmers only show minor alterations compared to naïve volunteers to suggests adaptation [36,37]. It is possible that adaptation in animal models may be induced following exposures shorter than 8 hours.

Because we found high concentrations of endotoxin in the barn air, it may be central protagonist in initiating lung inflammation in the exposed animals. These data show that rat lung responses to the barn air are similar to those in humans and that rat may be a reliable model to simulate lung dysfunction in humans following exposure to the barn air [33].

## In vitro studies on the effects of swine barn dust

Several researchers have shown the inflammatory potential of swine barn dust or air in many *in vitro *experiments [38-40]. These in vitro studies are a valuable tool to understand the effects of swine barn air or dust on a variety of lung cells. These studies provide an opportunity to control various variables to facilitate identification of cellular and molecular pathways that regulate response to swine barn air. Swine dust induces release of IL-8 in normal human bronchial epithelial cells, human pulmonary epithelial carcinoma cell line (A549) and in human alveolar macrophages [38]. Swine dust is almost as potent as lipopolysaccharides in stimulating cytokine release from alveolar macrophages in vitro [39]. Recent data showed that swine barn dust activates protein kinase C to induce secretion of IL-8 and IL-6 from airway epithelial cells and promotes adhesion of lymphocytes through upregulation of ICAM-1 [40,41]. Swine barn dust can also directly activate T-lymphocytes [42]. These data show importance of performing in vitro studies along with human and experimental animal investigations to further our understanding of lung responses to pig barn air.

## Conclusion and future studies

The data from animal and human studies show that barn air can induce lung dysfunction. Recent data from animal studies and from in vitro studies have started to elucidate mechanisms of lung dysfunction induced following exposure to the barn air. However, many questions remain unanswered. One of the central questions relates to precise and relative contributions of various toxic molecules in the barn air to lung dysfunction. The endotoxin is the foremost toxic agent in the barn air. The role of endotoxin in barn air induced lung dysfunction can be assessed through the use of mice that lack a functional Toll-like receptor-4. Second logical experiment is the physical and biochemical characterization of the dust particles in the barn air. Specifically, we need to know if the barn air contains dust particles which are less then 100 nm in size because particles of this size are believed to provoke a vigorous cardiopulmonary response [43,44]. The biochemical characterization of the particles would reveal if particles are used as Trojan horse to carry endotoxins and other toxic molecules into the lungs.

## Competing interests

The authors declare that they have no competing interests.

## Authors' contributions

Both the authors contributed equally in the preparation of this review.

## References

[B1] (2006). World Pork Meat and Swine Trade Overview. http://www.fas.usda.gov/dlp/circular/2005/05-04LP/porkoverview.pdf.

[B2] (2006). Hog Statistics.

[B3] (2001). Farm operators by farm type, by province ( 2001 Census of Agriculture) ( Canada). http://www40.statcan.ca/l01/cst01/agrc22a.htm.

[B4] Cole D, Todd L, Wing S (2000). Concentrated swine feeding operations and public health: a review of occupational and community health effects. Environ Health Perspect.

[B5] Wenger I (1999). Air Quality and Health of Career Pig Barn Workers. Advances in Pork Production.

[B6] Wenger II, Ouellette CA, Feddes JJ, Hrudey SE (2005). The design and use of the Personal Environmental Sampling Backpack (PESB II) for activity-specific exposure monitoring of career pig barn workers. J Agric Saf Health.

[B7] Asmar S, Pickrell JA, Oehme FW (2001). Pulmonary diseases caused by airborne contaminants in swine confinement buildings. Vet Hum Toxicol.

[B8] Donham KJ, Popendorf W, Palmgren U, Larsson L (1986). Characterization of dusts collected from swine confinement buildings. Am J Ind Med.

[B9] Donham KJ, Popendorf WJ (1985). Ambient levels of selected gases inside swine confinement buildings. Am Ind Hyg Assoc J.

[B10] Cormier Y, Israel-Assayag E, Racine G, Duchaine C (2000). Farming practices and the respiratory health risks of swine confinement buildings. Eur Respir J.

[B11] Zejda JE, Hurst TS, Rhodes CS, Barber EM, McDuffie HH, Dosman JA (1993). Respiratory health of swine producers: Focus on young workers. Chest.

[B12] Schenker MB, Christiani D, Cormier Y, Dimich-Ward H, Doekes G, Dosman JA, Schenker MB (1998). Respiratory health hazards in agriculture.

[B13] Iversen M, Dahl R, Korsgaard J, Hallas T, Jensen EJ (1988). Respiratory symptoms in Danish farmers: an epidemiological study of risk factors. Thorax.

[B14] Zejda JE, Barber E, Dosman JA, Olenchock SA, McDuffie HH, Rhodes C, Hurst T (1994). Respiratory health status in swine producers relates to endotoxin exposure in the presence of low dust levels. J Occup Med.

[B15] Iversen M, Kirychuk SP, Drost H, Jacobson L (2000). Human health effects of dust exposure in animal confinement buildings. J Agric Saf Health.

[B16] Donham KJ, Zavala DC, Merchant JA (1984). Respiratory symptoms and lung function among workers in swine confinement buildings: a cross-sectional epidemiological study. Arch Environ Health.

[B17] Dosman JA, Graham BL, Hall D, Pahwa P, McDuffie HH, Lucewicz M, To T (1988). Respiratory symptoms and alterations in pulmonary function tests in swine producers in Saskatchewan: results of a survey of farmers. J Occup Med.

[B18] Bongers P, HJouthuijs D, Remijn B, Brouwer R, Biersteker K (1987). Lung function and respiratory symptoms in pig farmers. Br J Ind Med.

[B19] Haglind P, Rylander R (1987). Occupational exposure and lung function measurements among workers in swine confinement buildings. J Occup Med.

[B20] Zhou C, Hurst TS, Cockcroft DW, Dosman JA (1991). Increased airway responsiveness in swine farmers. Chest.

[B21] Larsson K, Eklund AG, Hansson LO, Isaksson BM, Malmberg PO (1994). Swine dust causes intense airways inflammation in healthy subjects. Am J Respir Crit Care Med.

[B22] Schwartz DA, Donham KJ, Olenchock SA, Popendorf WJ, van Fossen DS, Burmeister LF, Merchant JA (1995). Determinants of longitudinal changes in spirometric function among swine confinement operators and farmers. Am J Respir Crit Care Med.

[B23] Kennedy SM, Christiani DC, Eisen EA, Wegman DH, Greaves IA, Olenchock SA, Ye TT, Lu PL (1987). Cotton dust and endotoxin exposure-response relationships in cotton textile workers. Am Rev Respir Dis.

[B24] Lorenz E, Jones M, Wohlford-Lenane C, Meyer N, Frees KL, Arbour NC, Schwartz DA (2001). Genes other than TLR4 are involved in the response to inhaled LPS. Am J Physiol Lung Cell Mol Physiol.

[B25] Michel O, Duchateau J, Sergysels R (1989). Effect of inhaled endotoxin on bronchial reactivity in asthmatic and normal subjects. J Appl Physiol.

[B26] Jagielo PJ, Quinn TJ, Qureshi N, Schwartz DA (1998). Grain dust-induced lung inflammation is reduced by Rhodobacter sphaeroides diphosphoryl lipid A. Am J Physiol.

[B27] Michel O, Kips J, Duchateau J, Vertongen F, Robert L, Collet H, Pauwels R, Sergysels R (1996). Severity of asthma is related to endotoxin in house dust. Am J Respir Crit Care Med.

[B28] Malmberg P, Larsson K (1993). Acute exposure to swine dust causes bronchial hyperresponsiveness in healthy subjects. Eur Respir J.

[B29] Kirychuk SP, Senthilselvan A, Dosman JA, Zhou C, Barber EM, Rhodes CS, Hurst TS (1998). Predictors of longitudinal changes in pulmonary function among swine confinement workers. Can Respir J.

[B30] Wang Z, Manninen A, Malmberg P, Larsson K (1998). Inhalation of swine-house dust increases the concentrations of interleukin-1 beta (IL-1 beta) and interleukin-1 receptor antagonist (IL-1ra) in peripheral blood. Respir Med.

[B31] Larsson BM, Palmberg L, Malmberg PO, Larsson K (1997). Effects of exposure to swine dust on levels of IL-8 in airway lavage fluid. Thorax.

[B32] Donham KJ, Leininger JR (1984). Animal studies of potential chronic lung disease of workers in swine confinement buildings.. Am J Vet Res.

[B33] Charavaryamath C, Janardhan KS, Townsend HG, Willson P, Singh B (2005). Multiple exposures to swine barn air induce lung inflammation and airway hyper-responsiveness. Respir Res.

[B34] Wang Z, Larsson K, Palmberg L, Malmberg P, Larsson P, Larsson L (1997). Inhalation of swine dust induces cytokine release in the upper and lower airways. Eur Respir J.

[B35] Larsson BM, Larsson K, Malmberg P, Palmberg L (2002). Airways inflammation after exposure in a swine confinement building during cleaning procedure. Am J Ind Med.

[B36] Israel-Assayag E, Cormier Y (2002). Adaptation to organic dust exposure: a potential role of l-selectin shedding?. Eur Respir J.

[B37] Palmberg L, Larssson BM, Malmberg P, Larsson K (2002). Airway responses of healthy farmers and nonfarmers to exposure in a swine confinement building. Scand J Work Environ Health.

[B38] Palmberg L, Larsson BM, Malmberg P, Larsson K (1998). Induction of IL-8 production in human alveolar macrophages and human bronchial epithelial cells in vitro by swine dust. Thorax.

[B39] Wang Z, Malmberg PO, Ek A, Larsson K, Palmberg L (1999). Swine dust induces cytokine secretion from human epithelial cells and alveolar macrophages. Clin Exp Immunol.

[B40] Romberger DJ, Bodlak V, von Essen SG, Mathisen T, Wyatt TA (2002). Hog barn dust extract stimulates IL-8 and IL-6 release in human bronchial epithelial cells via PKC activation. J Appl Physiol.

[B41] Mathisen T, von Essen SG, Wyatt TA, Romberger DJ (2004). Hog barn dust extract augments lymphocyte adhesion to human airway epithelial cells. J Appl Physiol.

[B42] Muller-Suur C, Larsson PH, Larsson K, Grunewald J (2002). Lymphocyte activation after exposure to swine dust: a role of humoral mediators and phagocytic cells. Eur Respir J.

[B43] Aktas Y, Yemisci M, Andrieux K, Gursoy RN, Alonso MJ, Fernandez-Megia E, Novoa-Carballal R, Quinoa E, Riguera R, Sargon MF, Celik HH, Demir AS, Hincal AA, Dalkara T, Capan Y, Couvreur P (2005). Development and brain delivery of chitosan-PEG nanoparticles functionalized with the monoclonal antibody OX26. Bioconjug Chem.

[B44] Costantino L, Gandolfi F, Tosi G, Rivasi F, Vandelli MA, Forni F (2005). Peptide-derivatized biodegradable nanoparticles able to cross the blood-brain barrier. J Control Release.

